# Complete abdominal aortic occlusion following seat-belt trauma: a proposal for early recognition

**DOI:** 10.1093/bjrcr/uaag015

**Published:** 2026-04-13

**Authors:** Antonio Galluzzo, Fabio Lampis, Chiara Esposito, Diletta Cozzi, Riccardo Ferrari, Michele Galluzzo, Vittorio Miele

**Affiliations:** Department of Experimental and Clinical Biomedical Sciences “Mario Serio”, University of Florence, Florence 50127, Italy; Department of Radiology, Duilio Casula University Hospital, Cagliari 09121, Italy; Department of Medical, Surgical and Neuro Sciences, Diagnostic Imaging, University of Siena, Siena 53100, Italy; Department of Emergency Radiology, Careggi University Hospital, Florence 50127, Italy; Department of Emergency Radiology, San Camillo-Forlanini Hospital, Rome 00100, Italy; Department of Emergency Radiology, San Camillo-Forlanini Hospital, Rome 00100, Italy; Department of Experimental and Clinical Biomedical Sciences “Mario Serio”, University of Florence, Florence 50127, Italy; Department of Emergency Radiology, Careggi University Hospital, Florence 50127, Italy

**Keywords:** aorta, trauma imaging, early diagnosis, emergency radiology

## Abstract

Post-traumatic abdominal aortic occlusion (PTAO) is an extremely rare but potentially fatal consequence of high-energy blunt trauma, particularly in the setting of seat-belt injury. While thoracic aortic injuries are more commonly reported, complete occlusion of the abdominal aorta remains poorly documented, with only a few case reports available in the literature. We present two cases of seat-belt-associated PTAO, both evaluated with contrast-enhanced computed tomography (CECT), highlighting the importance of early recognition and tailored imaging protocols in polytrauma scenarios. In both cases, the aortic injury was located at the level of L3, anatomically aligned with vertebral fractures, supporting a direct traumatic mechanism. One patient underwent emergent surgical repair but succumbed to complications, while the other was successfully treated with an endovascular approach and remained stable at one-year follow-up. Key diagnostic features included sudden absence of aortic opacification, absence of vascular calcifications, and lack of collateral circulation, helping distinguish acute PTAO from chronic conditions such as Leriche syndrome. Our findings emphasize the need for radiologists and emergency clinicians to maintain a high index of suspicion for PTAO in patients with seat-belt injuries and vertebral fractures. Prompt diagnosis using CECT can guide urgent therapeutic decisions and improve patient outcomes. This report adds to the limited literature on abdominal aortic injury following blunt trauma and proposes practical imaging criteria for early detection.

## Introduction

Post-traumatic complete occlusion of the aorta (PTAO) is a rare and scarcely documented condition. Most acute aortic occlusions occur in high-risk patients and result from in situ thrombosis in an atherosclerotic aorta (64.1%), saddle embolism (21.3%), or occlusion of vascular devices (14.7%).^1–3^ Aortic injury (AI) caused by seat-belt trauma—referred to as “seat belt aorta”—falls within the broader seat belt syndrome, which also includes visceral injuries, Chance vertebral fractures, and the superficial “seat belt sign”.^4^

Seat belt aorta may manifest as thrombosis, dissection, embolization, or aneurysm, typically due to aortic compression between the belt and spine during high-impact deceleration. Early diagnosis is crucial and should be considered in all patients with high-energy blunt trauma and signs of abdominal injury or associated visceral damage. In complex polytrauma cases, contrast-enhanced computed tomography (CECT) enables rapid and systematic assessment of patient status, including the identification of aortic injury (AI).^5^ Management depends on injury severity and hemodynamic status, ranging from open surgery to endovascular repair.^6–10^ A review confirmed an association between PTAO, thoracolumbar spine (TLS) trauma, and Chance fractures.^11^ Only a few cases of PTAO diagnosed by CECT have been reported.^12–14^ We present two additional cases of seat belt aorta and PTAO documented on CECT, along with a literature review and a proposal for prompt recognition.

## Case 1

**Figure 1:**
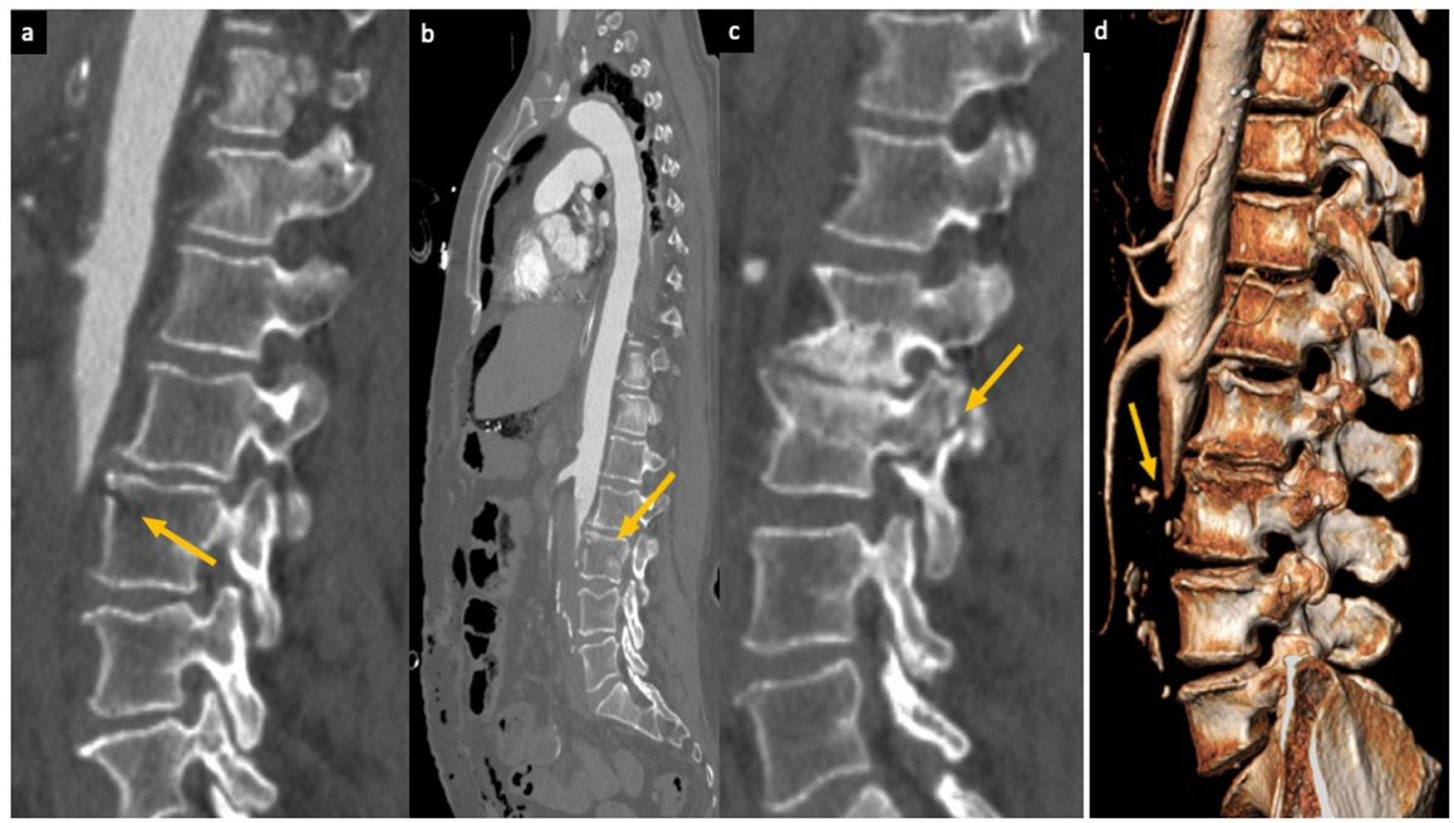
Sagittal MPR (a, b, c), and volume-rendering (d) CT images of the lumbar spine and abdominal aorta. Images in a, b, c demonstrate a horizontal fracture line at the level of L3, involving all three columns of the vertebral body (anterior, middle, and posterior), consistent with a Chance-type flexion-distraction injury. The same images show that the abrupt lack of contrast opacification in the abdominal aorta begins precisely at the level of the vertebral fracture, suggesting a traumatic origin of the vascular injury. Image (d), obtained with 3D volume rendering, clearly depicts the sudden interruption of the aortic lumen directly aligned with the fracture site at L3.

**Figure 2:**
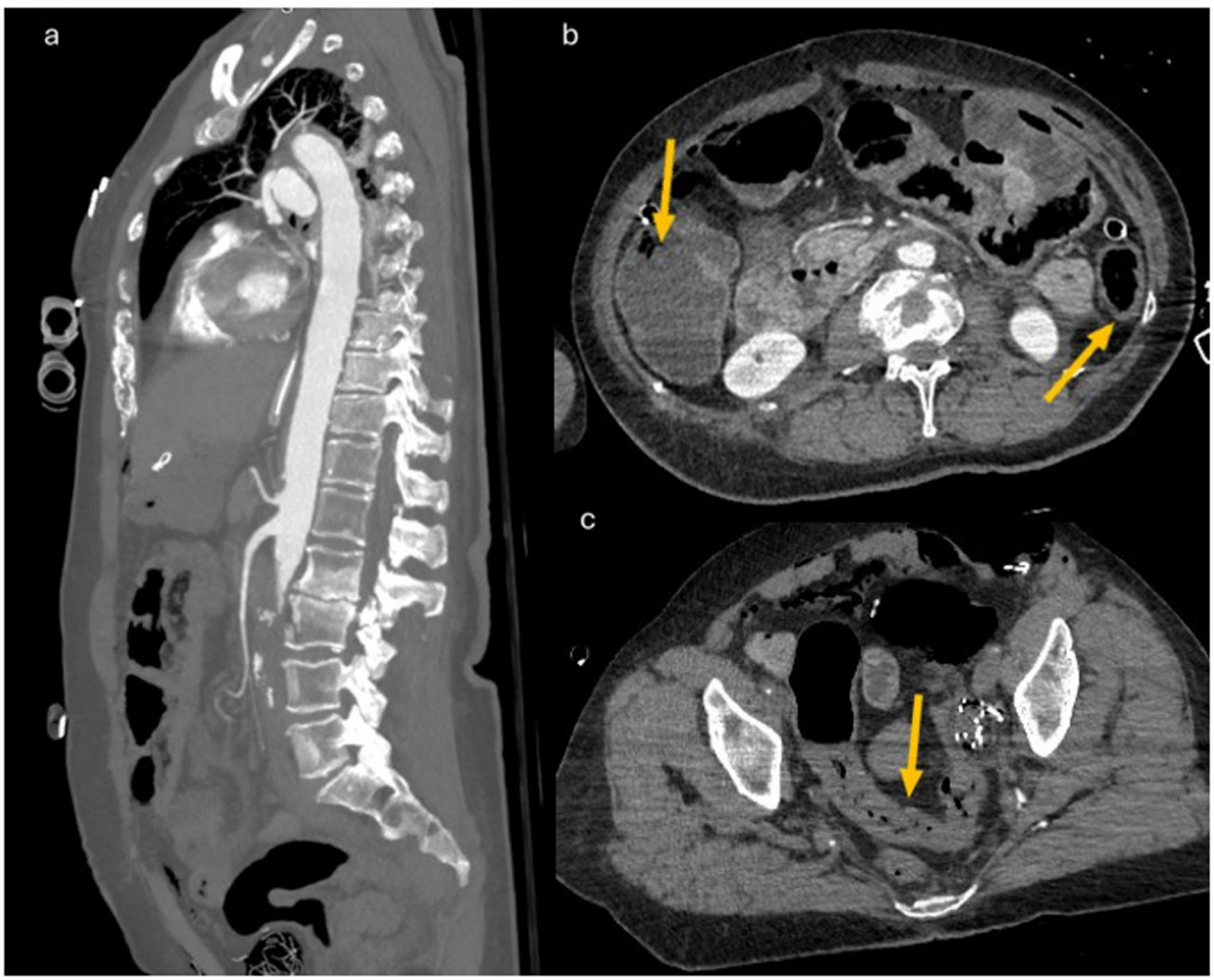
Multiplanar and axial CT findings of intestinal ischemia secondary to acute inferior mesenteric artery occlusion. (a) Sagittal MPR reconstruction in the arterial phase shows normal opacification of the superior mesenteric artery (SMA), while the inferior mesenteric artery (IMA) is not visualized, its origin being located within the segment of thrombosed abdominal aorta. Axial venous-phase images demonstrate preserved enhancement of the ascending colon, supplied by the SMA (arrows in b), contrasted with hypoenhancement and mural thickening of the descending and sigmoid colon, consistent with intestinal ischemia in the IMA territory (arrows in c). Signs of postoperative pneumoperitoneum are also visible, related to the recent abdominal surgery.

A 53-year-old woman with no significant medical history was involved in a high-energy motor vehicle collision. She was seated in the rear of a stationary car that was violently struck and propelled against a fixed barrier. The patient was wearing a seatbelt at the time of impact and arrived at the emergency department around 08:30 AM. On initial evaluation, she presented with hypotension (BP 80/60 mmHg), tachycardia (HR 110 bpm), and an oxygen saturation of 97% under mechanical ventilation. She had been intubated pre-hospital due to altered consciousness. No external bleeding, pelvic instability, or major limb deformities were noted during the primary survey. According to the institutional trauma protocol, an Extended Focused Assessment with Sonography for Trauma (E-FAST) was performed, which revealed a large hemoperitoneum and altered splenic echogenicity, suggestive of high-grade injury. No pneumothorax was identified. Due to the positive E-FAST and persistent hemodynamic instability, the patient was taken emergently to the operating room. Intraoperative findings confirmed a grade V splenic rupture and multiple hepatic lacerations. A splenectomy and hepatic packing were performed. The procedure lasted approximately 40 minutes, after which a post-operative contrast-enhanced CT (CECT) was performed for further trauma assessment. The scan included pre-contrast, arterial, and venous phases, with multiplanar reconstructions (MPR) and maximum intensity projections (MIP) for vascular evaluation. CECT confirmed the surgical findings and revealed a complete absence of contrast opacification in the abdominal aorta, starting at the level of L3 and extending to the iliac bifurcation and both common femoral arteries. At the same level, a three-column fracture of the L3 vertebral body was identified, consistent with a Chance-type flexion-distraction injury (Figure 1). Additional findings included hypoenhancement and mural thickening of the descending and sigmoid colon, suggestive of early bowel ischemia, corresponding anatomically to the thrombosed origin of the inferior mesenteric artery (Figure 2). The patient was immediately transferred to the hybrid operating suite for a two-step intervention: prosthetic replacement of the thrombosed abdominal aorta and left hemicolectomy with colostomy, due to ischemia of the descending colon. Although no gross infarction was noted during the initial laparotomy, CT findings and worsening laboratory values prompted a second-look exploration, confirming segmental colonic necrosis. At approximately 11:00 AM—an hour after CT acquisition and less than three hours post-trauma—the patient developed acute bilateral lower limb ischemia, with cold, cyanotic extremities and absent peripheral pulses. These findings confirmed the diagnosis of acute post-traumatic aortic occlusion, ruling out chronic etiologies. No further imaging was obtained. Despite aggressive surgical and resuscitative efforts, the patient experienced cardiac arrest and died within hours of the intervention.

## Case 2

**Figure 3:**
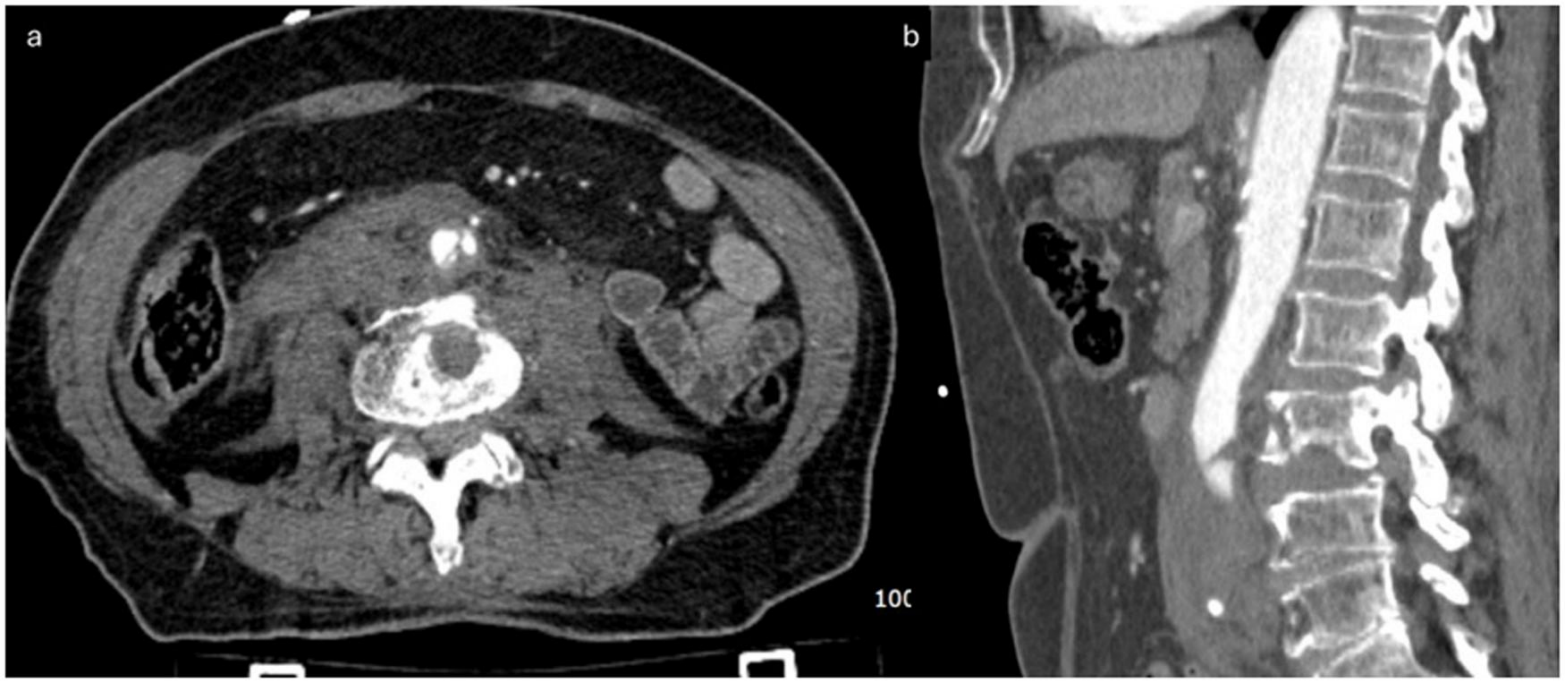
CT at admission in Emergency Department. (a) Axial arterial phase CT scan and (b) sagittal MPR reconstruction show complete lack of opacification of the abdominal aorta beginning at the level of L3. A fracture of the anterior vertebral body of L3 is clearly visible. In both planes, an intimal dissection flap is appreciable just proximal to the point of aortic occlusion, supporting the diagnosis of traumatic aortic dissection with subsequent intraluminal thrombosis.

**Figure 4:**
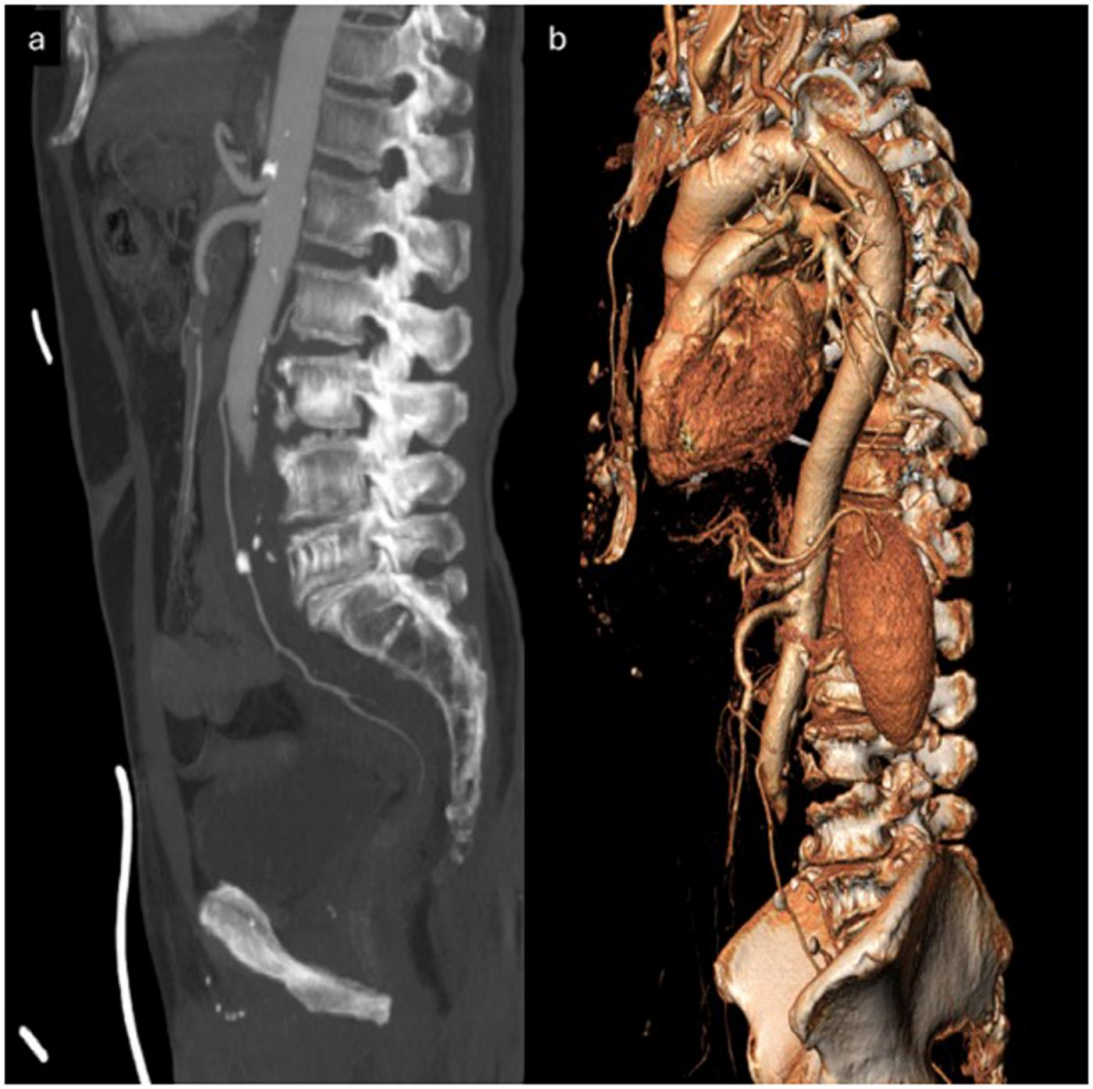
(a) Sagittal MIP reconstruction and (b) VRT reconstruction show the origin of both the superior and inferior mesenteric arteries proximal to the site of traumatic aortic occlusion at L3 level.

**Figure 5:**
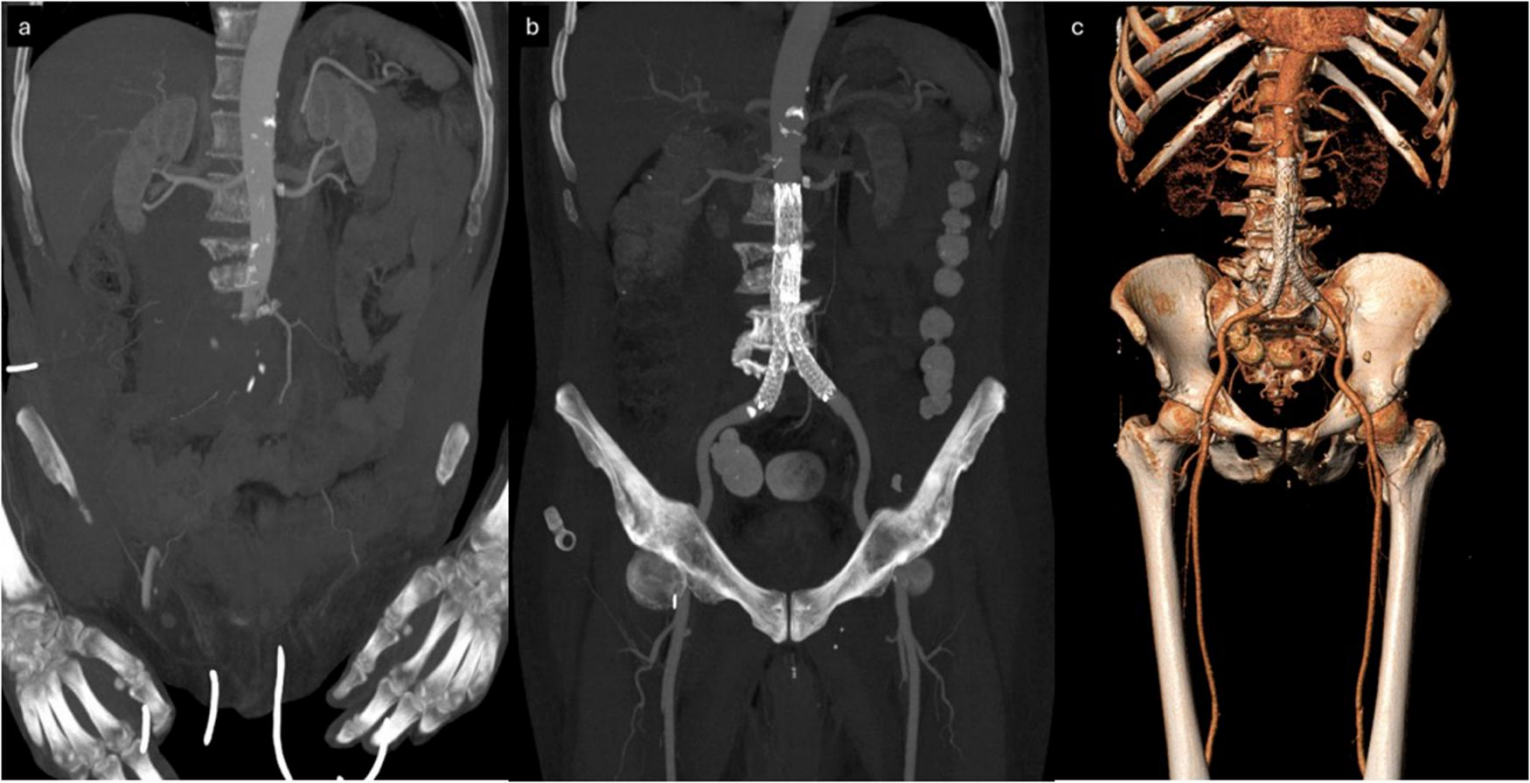
Coronal MIP reconstructions. (A) Initial CT at admission shows absence of opacification of the abdominal aorta distal to the level of L3, with poor visualization of the iliac or femoral arteries. (B) Post-operative CT following endovascular repair with aortic endograft demonstrates restored opacification of the abdominal aorta and excellent visualization of the iliac arteries, including both internal and external branches, as well as the common femoral arteries and their distal runoff. Image in c is a VRT of a follow-up CT at 10 days, demonstrating correct positioning of an aorto-bi-iliac endograft with complete restoration of vascular opacification of the abdominal aorta, its major branches, and the arterial circulation of both lower limbs.

A 70-year-old man with no significant medical history, except for a remote history of intravenous drug use, was involved in a high-speed frontal collision with a heavy truck while driving a high-performance vehicle. He was wearing a seatbelt at the time of impact and required prolonged extrication. The patient was airlifted and arrived at our trauma center at approximately 5:00 PM. On admission, he presented with a low Glasgow Coma Scale (GCS) score and had already been intubated at the scene. Initial vital signs included a blood pressure of 125/70 mmHg and an oxygen saturation of 98% under mechanical ventilation. As per institutional trauma protocol, an E-FAST exam was performed, which was negative for intra-abdominal free fluid and pneumothorax. A whole-body CECT scan revealed a complete absence of contrast opacification in the abdominal aorta, beginning at the level of L3, where an isolated anterior column fracture of the vertebral body was noted. The middle and posterior columns were spared, and no other spinal fractures were present. The location of the aortic injury at L3—a transition zone between fixed and mobile aortic segments—further supported a mechanism of blunt deceleration trauma, classically seen in seatbelt-related injuries. Importantly, there was no evidence of chronic aortoiliac occlusive disease. Arterial wall calcifications were minimal and age-appropriate. No collateral vascular networks were observed in the epigastric, mesenteric, or intercostal territories, and the patient’s hemodynamic stability further supported an acute traumatic occlusion rather than a chronic process (Figures 3 and 4). Peripheral pulses were absent on examination, consistent with acute lower limb ischemia. Over the next 90 minutes, the patient developed worsening ischemic signs in both lower extremities, including progressive coldness and cyanosis of the feet. He was urgently transferred to the angiographic suite for endovascular treatment. Digital subtraction angiography confirmed the presence of a traumatic aortic dissection with intraluminal thrombosis. A thoracoabdominal endovascular stent graft was successfully deployed via percutaneous femoral access, restoring antegrade perfusion. The procedure was uncomplicated, with immediate recovery of peripheral pulses and normalization of lower limb perfusion. No other major thoracic or abdominal injuries were identified. Cranial CT revealed a small subdural hematoma and minor bilateral frontal contusions. Additional injuries included bilateral tibial fractures. The patient was admitted to the ICU and had an uneventful postoperative course. He subsequently underwent orthopedic fixation of the tibial fractures and initiated an intensive rehabilitation program, focusing on both neurological recovery and motor function. A CT angiography performed 10 days post-intervention confirmed correct stent placement, absence of endoleak, and restored distal arterial perfusion. Notably, the origins of the superior and inferior mesenteric arteries were located proximal to the occlusion site, likely accounting for the absence of bowel ischemia. At 1-year follow-up, the patient remained alive, in good health, and with preserved functional status and quality of life (Figure 5).

## Discussion

We presented two cases of seat-belt–associated aortic injury, also referred to as “seat belt aorta,” complicated by post-traumatic abdominal aortic occlusion (PTAO). Based on a thorough literature review, no previous reports have included CT imaging of such catastrophic cases of PTAO. In polytrauma patients, aortic injuries carry a mortality rate of up to 80–90%.^15^^,^^16^ These injuries typically result from rapid acceleration–deceleration forces, often combined with anteroposterior or lateral impacts, which stretch the aorta and apply shearing and torsional stresses, especially at fixed anatomical segments such as the aortic isthmus, root, and diaphragmatic hiatus. Another proposed mechanism is the “osseous pinch,” where the aorta is compressed between the sternum and spine. The water-hammer effect, caused by a sudden rise in intraluminal pressure, may also contribute to damage of the aortic wall, producing a range of acute injuries from intimal tears to complete transections.^17–19^ Aortic injuries are classified into minimal (MAI) or significant (SAI), depending on severity. Traumatic abdominal aortic injury (TAAI), although less common than thoracic involvement, accounts for approximately 5% of cases and can reach a mortality rate of 75% when untreated.^20–22^ The infrarenal abdominal aorta is particularly vulnerable because of its anatomical fixation against the vertebral column. Lap belt compression may transmit shearing forces from the abdomen to the spine, causing vascular damage in this region. Both direct and indirect signs can be detected on CT imaging.^17^ Direct signs include intimal flaps, mural hematomas, pseudoaneurysms, and active contrast extravasation, while retroperitoneal hematomas are a common indirect finding. Thoracolumbar spine (TLS) fractures occur in nearly 9% of blunt trauma cases, with Chance-type fractures particularly relevant.^9^^,^^23^^,^^24^ These results from flexion-distraction injuries where the center of rotation lies anterior to the anterior longitudinal ligament, subjecting all posterior structures—including the aorta—to distraction forces. Hyperextension mechanisms may also exert similar forces, producing “reverse Chance” injuries. It is worth noting that vertebral fractures with retroperitoneal hematoma occur more frequently without aortic involvement, and not all aortic opacification defects are due to trauma. Chronic occlusive disease, particularly Leriche syndrome (LS), must be considered in the differential diagnosis.^25–28^ LS is an atherosclerotic condition affecting the abdominal aorta and iliac arteries and is typically associated with cardiovascular risk factors such as hypertension, hyperlipidemia, diabetes, smoking, male sex, and advanced age. While LS may cause symptoms like lower limb claudication, erectile dysfunction, and weak femoral pulses, some patients remain asymptomatic due to the development of collateral circulation.

**Figure 6:**
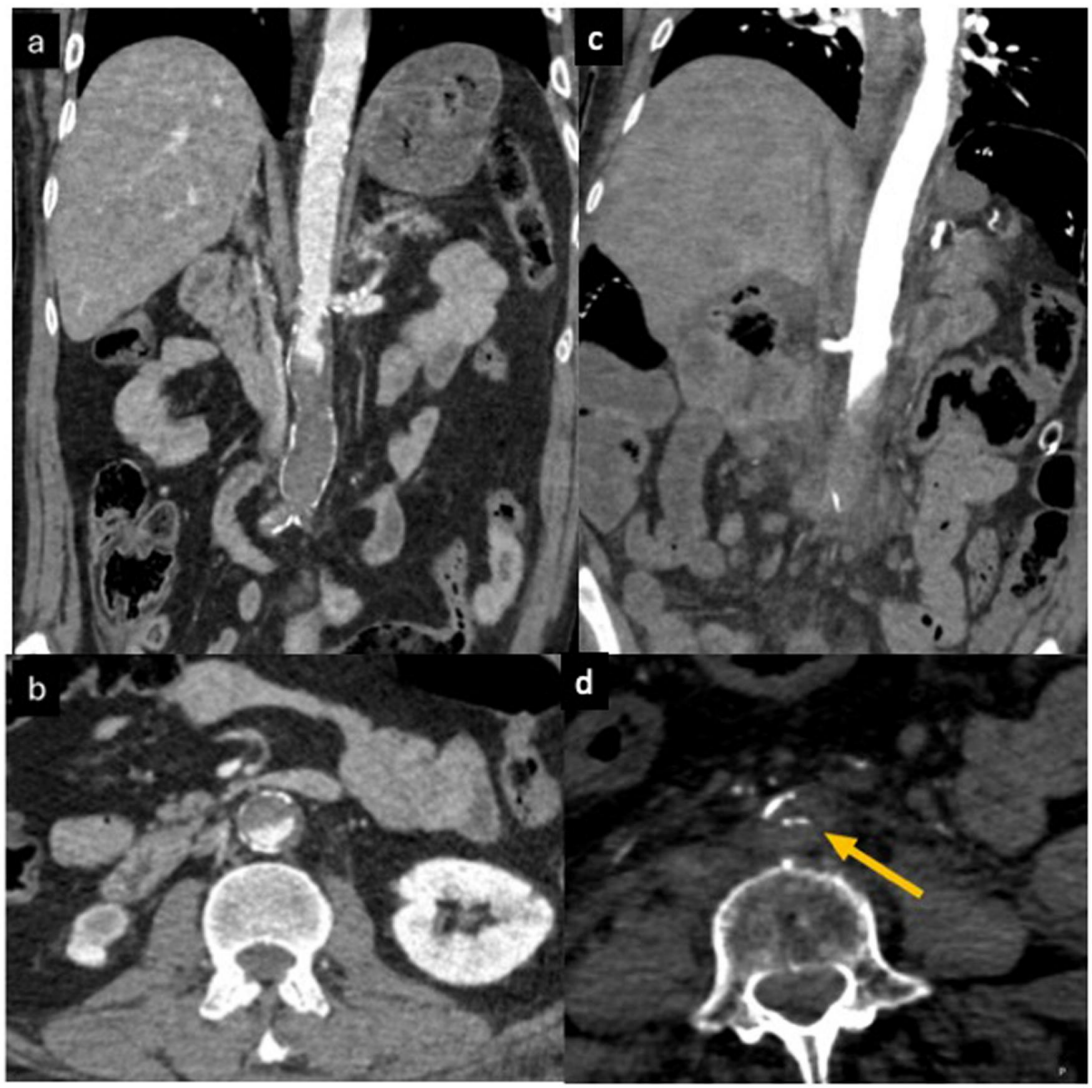
Comparison between chronic aortoiliac occlusive disease (A coronal–B axial) and acute traumatic aortic injury (C coronal–D axial). In figures A–B the patient with chronic Leriche syndrome shows diffuse aortic wall calcifications, particularly evident along the abdominal aorta. A mild pre-bifurcation ectasia is also visible, suggestive of chronic aortic wall weakening. The axial scan demonstrates circumferentially arranged calcifications outlining the normal course of the vessel lumen. In images in C–D corresponding images from our patient demonstrate an abrupt aortic occlusion secondary to traumatic dissection and thrombosis. Only minimal mural calcifications are visible, and notably, some calcific fragments are displaced into the aortic lumen on the axial scan (arrow in D), consistent with endoluminal migration of intimal calcifications—a finding highly suggestive of traumatic intimal disruption, rather than chronic arteriopathy.

In the first case, the diagnosis of PTAO over LS was supported by the absence of cardiovascular risk factors, the lack of diffuse vascular calcifications, and the complete absence of collateral circulation, even in the setting of hypotension. Most notably, calcifications at the site of aortic occlusion were displaced into the lumen, suggesting acute intimal disruption (Figure 6). This pattern differs significantly from the circumferential peripheral calcifications typically seen in chronic atherosclerosis. These features, along with the anatomical overlap between the fracture and the site of vascular occlusion, pointed toward a shared traumatic mechanism—likely acute hyperflexion transmitted via the seat belt. The absence of collateral vessels was a key radiologic clue in differentiating PTAO from LS. Unfortunately, the outcome was fatal due to irreversible bowel ischemia.^29^^,^^30^ The second case, in contrast, is the first known instance in the literature of PTAO assessed with both pre- and post-operative CECT. The patient’s survival was likely due to the absence of mesenteric ischemia and the timely endovascular intervention.

In conclusion, we reported two exceptionally rare cases of post-traumatic abdominal aortic occlusion that may contribute to facilitating early recognition of this condition. Given the high lethality of this entity when unrecognized or untreated, prompt imaging diagnosis is critical. Increased awareness of its radiological features may be particularly useful in clinical settings where trauma expertise is limited, potentially improving detection and management of these devastating vascular injuries.

## Data Availability

Written informed consent was obtained from the patient(s) for publication of this case review, including accompanying images. Appropriate consents, permissions and releases were obtained and retained by the author and are available from the corresponding author on reasonable request.
